# Youth Serial Killers: Psychological and Criminological Profiles

**DOI:** 10.3390/ijerph19095359

**Published:** 2022-04-28

**Authors:** María Elena García-Baamonde, Macarena Blázquez-Alonso, Juan Manuel Moreno-Manso, Eloísa Guerrero-Barona, Mónica Guerrero-Molina

**Affiliations:** Department of Psychology, University of Extremadura, 06006 Badajoz, Spain; mgarsan@unex.es (M.E.G.-B.); mablazqueza@unex.es (M.B.-A.); eloisa@unex.es (E.G.-B.); monicagm@unex.es (M.G.-M.)

**Keywords:** serial killers, children, youth, profile, psychopathy

## Abstract

Serial murder is a specific type of violent crime that falls into the crime category of multicide. According to the nomenclature of the Federal Bureau of Investigation’s *Violent Crime Classification Manual* and Academic Researchers for the Classification of Violent Crimes, most serial killers are adults. However, serial murder is also committed by young people, although to a lesser extent. Young serial killers are a topic of relevance in areas such as psychology, criminology, and the justice system. Given that the study of the variables that could be the basis of such multicide criminality is not conclusive, the need for further research is evident. The homicides perpetrated by children and young people point to a social panorama that is alarming due to their young age. This issue is prevalent enough to conduct a review. The performed review concludes the importance of psychosocial factors to better understand the process by which children and young people commit crimes as serious as serial murders. The scope of the problem of serial murders perpetrated by minors is controversial because it often depends on how the number of real cases is counted. Although official statistics indicate the low prevalence of juvenile serial killers, childhood is a period in which antisocial behaviour can have its beginning. Some authors consider that it is not uncommon for the first murder of this type to occur in adolescence. It is important to consider psychopathy as an influential factor in the various forms of serial criminal conduct committed by children and young people. The research works consulted provide evidence of the special relevance of psychopathy in the generation of serious juvenile delinquency.

## 1. Introduction

The phenomenon of serial murders occupies a unique place in the field of Criminology and the criminal justice system, especially when the perpetrators of this and other kinds of violent crimes are underage. In this sense, we use the terms child, juvenile and youth without distinction, as in the Convention on the Rights of the Child [1], to refer to those under the age of 18 when which they committed homicide.

In addition to the difficulties that this supposes for criminal investigation by the police and judicial systems, serial murder cases attract excessive attention from the media, mental health experts, the academic world, and the general public. This social, media and professional impact is even worse when the serial murders are perpetrated by either young people or women, since reality is greatly softened in the case of these populations, the more unpleasant aspects of the crime tending to be mitigated [2].

Furthermore, much of the lack of understanding of the phenomenon of serial murders, especially in the case of underage perpetrators, is surrounded by a halo of media sensationalism that usually arises from a question about the mind of serial killers asked and debated across numerous articles and opinion columns: are serial killers born or made? Added to this is another question of a similar nature, which refers to the popular idea of serial killers as predators, monsters, or devils [3]. In this sense, if this sensationalist question had to be settled, the conclusion would be obvious: serial killers are predators only in the sense that their methods of attack are very similar to those of predatory animals—they stalk, attack, and kill [4]. On the other hand, labelling them as monsters or devils, however abominable and indescribable the acts of such individuals may be, is only part of the media circus surrounding such cases at both judicial and social levels [5], and contributes to the media-influenced collective mentality that spares no detail [6] and which even, inexplicably, makes celebrities of such people [7].

Nevertheless, the generally badly characterised film image of the typical murderer who creates a plan of action and chooses their victim is ostensibly false in the case of most juvenile homicides. Only in very few cases (mainly serial killers, mass murderers and itinerants) does it respond to reality [8].

Putting aside the speculations of the media world, only empirical works of research and academic books that deal rigorously with this delicate and singular subject were considered in this review on serial murders perpetrated by children and youths. In this review, the problem of children and youths who commit homicide or murder, but not serially, was first reviewed to differentiate them from those young people who are serial killers.

Secondly, the concept and characterization of serial murders were defined as a form of multicide and as a specific type of violent crime. According to the conceptualization given by both the FBI’s *Violent Crime Classification Manual* and various researchers, the great majority of serial killers are white males aged between 25 and 35. Nevertheless, serial murder is also committed by youths, although to a much lesser extent.

Thirdly, the analysis of the psychological and criminological profiles of children and youths who had committed serial murders allowed us to characterize them with a series of psychosocial aspects and criminal motivations. In addition, it is also important to stress the need to consider other types of serial criminal conduct in which some young people become involved, and which can be considered as forerunners to the later perpetration of serial murders. Fourthly, the importance and role played by child–juvenile psychopathy in violent, criminal conduct and the perpetration of serial murders was explored.

The acquisition and development of aggressive and violent behaviour patterns, which may increase the risk of minors committing murder, respond to the joint action of multiple etiological factors (psychological, social, biological, environmental, etc.). Given that the study of the variables which might comprise the basis of such multicide criminality is inconclusive, the need for further investigation is evident. This review aims to provide the necessary knowledge to outline the therapeutic needs of serial killer children, and to deal with them through specific interventions.

## 2. Method

To achieve a better knowledge of the phenomenon of murder committed by youths, as well as to understand the extent and severity of this phenomenon, we carried out a scoping review of research articles written in various countries. 

Similarly, we reviewed and compared research works related to the concept and characterisation of serial killers. In addition, we analysed the said manifestations in youths to be able to understand their psychological and criminological profiles.

The information was obtained from the following databases: ScienceDirect, Scopus and PsycoINFO. For this review, publications were selected using the following descriptors: (serial killers AND youths) AND (juvenile delinquency AND psychosocial OR psychopathy factors). 

Concerning exclusion criteria, all those studies in which the said search descriptors were not present were discarded. Similarly, despite the inclusion of research papers related to other serial criminal behaviour patterns often carried out by young people, such as arson, cruelty to animals, or sexual crimes [9], those studies that referred to non-serial juvenile violence were excluded.

The year of publication of these research papers was not an exclusion criterion, given their scarcity.

## 3. Results

### 3.1. Youth Homicides and Non-Serial Killers: Extent and Severity of the Problem 

The extent and severity of the problem of serial murders committed by minors (children and youths) begins with homicide and murder of a non-serial nature, but equally violent. There are numerous cases of this type throughout the world, and they are widely documented.

Despite the terminology frequently used by the media, young delinquents are not monsters or beasts, and often have not committed previous acts of violence [10]. The analysis and investigation that this author carried out on 80 attempted or consummated homicides, perpetrated by youths, shows that a great number of these crimes occurred because they found it difficult to refuse to participate in something previously planned by a group of peers, so the criminal motivations were only partially due to the minor’s personality. An explanation can often be found in an accumulation of arbitrary circumstances, and in the way an extremely aggressive individual reacts to such circumstances [10].

In this sense, Lempp [10] studied the probable motives for homicides committed by young delinquents and situated them in psychic and environmental contexts for each case to contribute to the scientific study of the phenomenon, evaluating the psychological, legal, psychiatric, and social aspects of each case.

Nevertheless, as mentioned previously, if the reality of these cases seems to be twisted, then what is the real extent of the problem? In this sense, the controversy and debate concerning the extreme violence of minors is widespread, since there are a diverse range of opinions and data about criminality in general among minors and young people. Rechea and Fernández [11] consider that the percentage of young people who commit this type of act is low.

The analysis of the participation of juveniles in homicides is still a serious problem in the USA, since between the mid-1980s and the start of the 1990s, there was an unprecedented growth in homicides perpetrated by youths [12]. The available data suggest that juveniles currently participate in more homicides than in previous generations [13]. 

Independently of the figures, the cases of juvenile homicide and murder cannot be underestimated, since the psychosocial and criminological reality of these cases is as particular as it is fatal for the victims.

Braga, Kennedy, Waring, and Morrison [14] stress the significance of homicides carried out by youths who belong to street gangs. It must be said that this criminal phenomenon is very particular, as its situational variables are different and should be studied separately from the rest of juvenile crime [15,16]. 

Due to all of the above, the psychosocial and criminological profile of youths who commit homicide and murder is not comparable to that of the common delinquent. In this sense, Lempp [10] warns that greater importance is not given to cases of child–juvenile homicide due to the generalized belief that “no-one would have believed them capable of such acts, precisely because they did not fit in any way the general image we have of a murderer”.

### 3.2. Serial Murders: Conceptual Limits and Main Characteristics

There is much controversy about the operational definition of serial murder [17]. However, in general, it can be considered a kind of multicide that can be defined as the repeated homicide of two or more persons [18], and with a cooling-down period between one crime and another [4].

The term serial killer was first coined to identify individuals who methodically murder a number of people within a period of time, although the motives, the victims and the methods vary from one serial killer to another [19].

Although the minimum number of victims considered necessary to define murders as serial murders is arbitrary, authors such as Egger [20] place the said minimum number as at least three victims. Similarly, although there is no single, generally accepted definition, Ferguson, White, Cherry, Lorenz, and Bhimani [21] coincide in pointing out that the essential element for defining a serial killer is the execution of three or more murders during multiple discreet events. Therefore, traditionally, a serial killer is defined as an individual who has murdered three or more victims [18].

However, it is important to point out that there are other types of homicide (for instance, terrorists, mass murderers, etc.) in which the perpetrators can murder more than three victims in their first killing spree, but who can then still become serial killers with a cooling-down period between one crime and another [22].

In any case, the aspect of the number of victims would have to be reconsidered, as there are murderers who only kill one person and are quickly arrested and imprisoned. Then, after studying their criminal behaviour patterns, it can be seen that there is a high probability that they would have killed again if they had not been caught and imprisoned [4]. 

The need to have a precise, working definition of a serial killer takes on greater importance when considering the process of generating the psychological and criminological profile of this type of individual, since there are many subtypes of serial killer. This gives rise to a variety of behaviour patterns and criminal motivations that reflect heterogeneous characteristics [23]. It is thus important to establish the psycho-criminological profile of these multicides on a differential level [24,25].

### 3.3. The Phenomenon of Serial Murders Committed by Minors

#### 3.3.1. The Psycho-Criminological Profiles of Child and Youth Serial Killers: Psychosocial Aspects and Criminal Motivations

Serial murder is a type of multicide predominantly perpetrated by male adults [22]. The cases of child and youth serial killers are, of course, much less frequent than adult cases [9]. From the academic and professional sphere of forensic psychiatry, Myers [26] studied six cases of serial killers who were under 18 years of age throughout the entire series of their crimes, identifying only six in the last 150 years, the majority from the USA. According to this author, at least two of the six serial killers began to commit murder in their adolescence, and most of their successive crimes occurred at somewhat older ages. 

According to Myers [26], each of these six young serial delinquents committed sexually motivated homicides as an expression of their aberrant erotic interest, which they later openly displayed at the crime scenes. The signs of sexual sadism and criminal characteristics reflect the behavioural profile of a more predatory type of violence (proactive or instrumental) than affective or reactive violence. In this sense, Myers [26] points out that these subjects preferred to use their own hands in the diverse methods and ways used to kill their victims, that is, cutting, stabbing, strangling and/or dismembering. Three of these children had problematic upbringings, yet, according to the reports, only one was physically abused by his father. The great majority of these juvenile serial killers showed signs of sexual sadism, a characteristic generally found in adult sexual serial killers [26].

Furthermore, this author states that “humans are not physiologically ‘wired’ to have sexual pleasure during the experience of significant anger” [26]. However, children usually explore their sexuality and the spectrum of pleasure/pain at an early age. Thus, according to this author, the physiological trait that traces the line between pleasure and pain is absent in these young serial killers, and it is this fact that could have given rise to the generalized conclusion that a serial killer’s behaviour is caused by psychological, social, and biological problems [26].

Some of these serial killers come from broken homes where they could not acquire a stable personality. Thus, they continually look to satisfy their desires through fantasies of domination and control [27]. Similarly, some of them were physically, sexually, and emotionally abused in childhood, and often such abuses were simultaneous [28]. 

Research into the impact of child abuse on violent behaviour has demonstrated that abuse and exposure to violence, in any of its multiple forms, is a very important factor for predicting criminal conduct [29]. The research of Dutton and Hart [30], carried out on males condemned for various crimes, suggests that those minors who were physically, sexually, and/or emotionally abused were three times more likely to act violently when adults. Nevertheless, Mitchell and Aamodt [31] state that, in the serial killer population, neglect experienced in childhood does not have a significant prevalence.

Myers [26] does not mention the important fact that the phenomenon of sexual pleasure/pain can be a learnt characteristic. In this sense, it had already been pointed out that “the blurring of pain/pleasure lines can occur during abuse, exposure to violence, or as psychological training (more often seen in socially accepted sexual masochism and slave/master social circles)” [32].

In any case, Myers concludes that “the murderers’ impulses to kill did not in the least decrease as they grew up; serial killers also fit into the category of ‘predator’” [26]. Due to the limited number of child serial killers, Myers [26] was unable to extrapolate the results of his study, and the idea of biological traits being the principal influence of serial killers requires more research to either prove or disprove it. In a later study on the psychological motivations of 12 sexual serial killers, this author and his team could only conclude that this type of individual expresses their positive feelings of sexual pleasure, and even euphoria (instead of anger or other unpleasant states of mind), through their criminal acts [33].

Although serial killings would seem to be a rare event, some authors suggest that it is difficult to determine the incidence and prevalence of this phenomenon [34]. Given that this form of violence has a strong social impact, and that it can appear to be the result of interacting biological, psychological, and sociological factors, research into this phenomenon should start in infancy [28].

As the cases of child and youth serial killers are much less frequent than those committed by adults, it is necessary to approach the studies of cases in this area differently to be able to advance our knowledge of such youths. Thus, an initiative to be recommended in this sense consists of more detailed research into other criminal serial behaviour patterns used by these youths. Such behaviour patterns as are set out below.

#### 3.3.2. The Role of Five Categories of Criminal Serial Behaviour Patterns Used by Children and Youths: Criminological History of Adult Serial Murders?

According to Myers and Borg [9], there are generally six categories of criminal serial behaviour patterns which are normally studied in the case of children and youths: (1) ‘generic’ juvenile delinquency (which provides an explicative framework for other violent or non-violent crimes); (2) crimes of firesetting; (3) crimes of cruelty to animals; (4) crimes of a sexual nature; (5) crimes of sadism; and (6) serial murders. According to these authors, “the first five categories do not represent all the possible forms of serial crimes committed by young people, and neither do they cover the more serious types or all the specific profiles” [9]. In this sense, they also point out that “a wider treatment of this area could include additional, less serious crimes which, are by nature serial, such as kleptomania” [9].

Based on a thorough review of the literature concerning the five categories of serial criminal behaviour patterns in children and youths, Myers and Borg [9] believe that many of them could be the precursors of later serial murders, by which they seem to be ‘testing things out’; for instance, how they feel upon setting fire to objects, houses, and forests, mistreating and killing animals, etc. Thus, the maximum expression of criminal behaviour would reside in the murder of one or more people. Some young people begin with other types of serial criminal behaviour patterns which serve as a ‘preparatory’ step. Does this mean that serial murder can be learned? Considering the application of the theory of social learning to acts of firesetting by children and youths, Singer and Hensley [35] found that the motivational patterns of the subjects suggested just that.

Although the research estimates that serial killers begin their criminal careers at 20 years of age, what is certain is that it is not uncommon for the first murder of this type of multicide to happen in adolescence. For example, Burgess, Hartman, Ressler, Douglas, and McCormack [36] found that 10 out of the 36 adult serial killers in their study, imprisoned for sexual homicide, had also previously committed murder when they were young. Similarly, it is important to mention that the behavioural indicators of sexual murder predict the presence of sadistic sexual fantasies in both delinquents and the general population [37].

Thus, the criminal conduct of serial sexual attacks is also an important factor to be considered when trying to better understand the criminological and psychosocial profile of serial killers [38,39]. This is because many of them perpetrate serial sexual homicides [40] with diverse patterns and motivations [41,42]. Similarly, crimes of firesetting committed by children and youths are also examples of criminal conduct that should be considered, as they appear in many cases of serial killers [43]. Juvenile sexual homicides seem, in many cases, to be correlates or criminological antecedents of adult serial killers [44].

### 3.4. Psychopathy, Violent Delinquency, and Serial Murder

#### 3.4.1. The Role of Child–Juvenile Psychopathy in Violence and Delinquency

It is certain that the first five categories of serial criminal conduct in children and youths studied by Myers and Borg [9] should be considered in research into both serial murders and attempts to understand the motivational psychology and criminal profiling of violent delinquents [45,46]. In addition to these types of serial criminal conduct, variable psychopathy is another relevant factor to be considered in these cases since these youths usually score high in psychopathy [47].

Psychopathy is a clinical–forensic construct that gives rise to serious problems in the affective, interpersonal, and behavioural domains, so much so that psychopaths can victimize and manipulate others apparently without their conscience being affected [48]. Psychopathy is characterized by a series of well-defined traits [49].

Psychopathy may appear in connection with two separate concepts: that which stresses the social factors or childhood experiences are the root of the disorder; and those who defend the view that the biological, psychological, and genetic elements are the biggest contributors to its appearance [50]. 

It is necessary to distinguish between subclinical psychopathy (or non-criminal psychopathy) and criminal psychopathy [2]. While subclinical psychopathy is studied in the general population [51,52,53,54,55,56], criminal psychopathy is studied in clinical–forensic contexts such as courts and prisons [8,50].

The study of psychopathy is useful for understanding seriously antisocial behaviour and violence among children and youths [57]. In this sense, juveniles who experience a variety of antisocial risk factors, such as mental health issues, upbringing problems, a history of substance abuse, or prolonged criminal participation, are characterised by a profound impulsiveness, emotional instability, and a total absence of guilt with respect to the execution of the crime [58]. The result is the appearance of psychopathic characteristics, which seem to be risk factors for persistent violence among minors [59].

Although there are numerous myths and unspecified associations surrounding psychopathy in relation to violence and criminality [60,61,62], what is certain is that research has found that criminal psychopathy is a risk factor as far as violence and recidivism are concerned [63,64,65]. Taking into account the marked antisocial behaviour of criminal psychopathy [66] and the characteristic of criminal versatility mentioned in the Psychopathy Checklist—revised (PCL-R) [50], the main difference between subclinical and criminal psychopathy lies in the concrete perpetration of a crime, of whatever kind, as the basic structure of personality and emotions is practically the same in both types of psychopaths, the criminal and subclinical [2].

Many traits of psychopathy begin to emerge in infancy [50] and are clearly identifiable and evaluable in childhood, adolescence, and youth [67]. However, the inclusion of the concept of psychopathy in the child–juvenile population is not without controversy. While Semel [68] stresses the limitations of the evaluation of juvenile psychopathy in clinical and forensic contexts, Forth, Bergstrøm, and Clark [69] clarify the necessity and implications of this evaluation of minors. Although professionals and doctors are still reticent about using the term ‘psychopath’ in the cases of children and youths [70], the scientific evidence in this respect cannot be denied due to its relation to criminal conduct in this population.

In light of the above, Dåderman [71] studied the personality traits present in adolescents diagnosed with severe behaviour disorders who showed psychopathy-related traits, e.g., searching for intense sensations, high impulsiveness, and low conformity, socialisation, and social desirability.

Glueck and Glueck [72,73,74] were the first to focus the subject of psychopathy on chronic and serious delinquents, some of whom were minors. These authors found that psychopathy was a useful variable for differentiating delinquents from non-delinquents. They described psychopathic delinquents as ostensibly destructive, antisocial, asocial, and less susceptible to therapeutic or educational efforts. Other characteristics include insensitivity towards social demands or towards others, a superficial emotionality, egocentrism, and a total lack of empathy [49], as well as, in many cases together with impulsive behaviour patterns, an absence of stress or anxiety concerning their lack of social adaptation, serious irresponsibility, and emotional poverty [48]. Young psychopaths seemed not to respond to any attempted treatment or rehabilitation, and they did not seem to be worried by their persistent criminal behaviour [72,73]. These authors observed that a psychopath was almost 20 times more common among their sample of delinquents than in the control group of non-delinquent subjects.

The relationship between child–juvenile psychopathy and chronic violent delinquency is currently still being studied. Salekin [75] studied a cohort of 130 children and youths to examine the effect of psychopathic personality on legal problems and life opportunities. This author found that psychopathy remained stable over the period the subjects were followed (four years), and the children with the highest scores of psychopathic traits in their early stages of life tended to maintain these scores later in adolescence. In addition, psychopathy is a significant predictor of both delinquency in general and various forms of violent delinquency. 

Current theories concerning crime (such as the situational prevention of crime) are based on the study of the circumstances in which these youths commit their crimes, rather than discovering the reasons why they committed them. Some authors have shown that it is possible to reduce the extent of the violence if the probability of detection increases and there is an effective response from the courts to dissuade young delinquents from enacting their violent behaviour patterns [14].

The effects of psychopathy on serious juvenile delinquency are more stable and persistent than the effects of the other 14 correlates of delinquency, including demographic characteristics, intelligence, previous delinquency and problems at school, parental factors, drug use, and delinquent companions, among others [75].

#### 3.4.2. The Role of Child–Juvenile Psychopathy in Serial Murder

The role played by psychopathy in the phenomenon of serial murders can be better understood if we look at its aetiology, which is not exempt from controversy. A great part of this controversy comes from the frequent generalization of the results of much research with common delinquents and non-serial but violent delinquents.

The problem concerning psychopathy lies in the fact that many of the results of research into the typical correlates of delinquency in general have been extrapolated to psychopathic delinquents, thus generating distorted images of psychopathy [62]. Similarly, the frequent and erroneous comparison of equating psychopathy with antisocial personality disorder (ASPD) becomes an additional problem because, while ASPD describes the common delinquent in general [8,49], most delinquents are not psychopaths, while not all psychopaths are delinquents [48,49]. Thus, in this respect, it should be stressed that the terms ASPD and psychopathy are often incorrectly used as synonyms [76]. ASPD is a kind of mental disorder (a personality disorder) included in psychiatric classifications, while psychopathy requires, according to the diagnostic criteria of Cleckley [49], a complete absence of any manifestation of a psychopathological nature.

The typical correlates of delinquency in general have been widely studied, and the most frequently studied variables or factors are diverse: gender, age, race, temperament, personality, self-control/self-regulation, school records, and family/parental factors [77]. Many antisocial delinquent youths present these and many other factors that are usually related to criminality. Although there are numerous clinical–psychiatric diagnoses associated with antisocial young people with behavioural problems [78,79], the great majority of young delinquents do not present evident and complete psycho-pathological syndromes, but simply serious problems of aggressivity and violence that respond to other explanations, such as psychosocial and criminological, rather than psychopathological [80,81]. To avoid pathologizing any type of criminal conduct, psychopathology is an area of study that should not adhere exclusively to the sphere of delinquency, as otherwise we could fall into the temptation of wrongly conceptualizing delinquency as a clinical disorder when it is not. Moreover, some delinquents present mental problems of diverse natures.

As for the specific role of child–juvenile psychopathy in serial murder, it is much easier to understand if the psychopathological approach is disregarded and we focus, more adequately, on the personality and motivations of this type of individual. In this sense, it is important to stress that one of the central, defining characteristics of psychopathy is the absence of empathy, reflected in a lack of consideration for and cruelty towards others, as well as a complete absence of remorse and feelings of guilt. Thus, if we pay attention to psychopathic personality and motivations, it is even possible to distinguish between simple homicides and multiple homicides through the presence or absence of psychopathy.

Woodworth and Porter [82] found that the characteristics of so-called simple homicides carried out in cold blood (instrumental and premeditated aggressors) depend on the psychopathy that characterizes them, emotional insensitivity, and a complete lack of remorse. These authors found that simple psychopathic homicides present a type of aggression that is predominantly instrumental (that is, premeditated, planned, ‘in cold blood’, motivated by an external objective and not preceded by any possible affective reaction), while simple non-psychopathic homicides present a type of aggression that is emotional (that is, not premeditated, reactive, or on the spur of the moment, motivated by an internal objective and preceded by some personal, emotional reaction).

‘Instrumental aggression’ is also present in most serial murders, and psychopathy plays a very important role. Many serial killers, in childhood and adolescence, present the typical traits of psychopathy [76] included in the serial criminal conduct categories of children and youths studied by Myers and Borg [9].

According to Morton [83], there is no generic profile for serial killers, who differ in many aspects, including their motivations for murder and their behaviour at the crime scene. However, certain common traits can be identified for some serial killers, including the desire for thrills, a lack of remorse, impulsiveness, the need for control, and abusive practices. These traits and behaviour patterns are consistent with psychopathy. The relationship between psychopathy and serial murders is particularly interesting. Not all psychopaths become serial killers, though serial killers usually possess some or many of the traits consistent with psychopathy. Psychopaths who commit serial murders put no value on human life and are extremely cruel in their interactions with their victims. This is particularly evident in serial killers with sexual motivations who repeatedly stalk, attack, and kill without remorse [83].

Nevertheless, it is also important to stress the fact that psychopathy alone does not explain the motivations of all serial killers [83], and neither does it explain the motivations of simple homicides. So why murder people, with or without psychopathy? This question is answered by Botelho and Gonçalves [84], who carried out a critical review of the literature on factors associated with homicides. Starting from a constructivist social perspective and ensuring that the factors related to homicides could be organized into four main categories, these authors observed that the acquisition and development of aggressive and violent behaviour patterns, which could provoke such a crime as homicide, had multi-factorial origins and implied multiple interconnections.

However, in the case of serial killers, it is necessary to stress the aspects of criminal motivations since they are specific in this type of multicide. In this sense, the concept of Perpetrator-Motive Research Design (PMRD), proposed by Vecchi, Van Hasselt, and Angleman [85], is of special relevance, as it deals with an analysis strategy that helps us understand the motivations, values and tactics of such violent delinquents as serial killers, serial rapists, paedophiles, youth murderers, arsonists, and cyber delinquents. This criminal investigation methodology could also help our understanding of the specific roles played by psychopathic traits in the criminal motivations of diverse types of violent delinquent, especially in the case of serial killers.

Although the absence of remorse is a principal characteristic of psychopathy found in adolescent delinquents and which ought to be investigated, along with other variables relevant at a theoretical and forensic level [86], it is not a characteristic which, of itself, can account for the diverse criminal motivations of the different types of violent delinquent when considering serial killers, who also have many other psychopathic traits. We thus believe that applying the PMRD methodology could help to clarify such questions and evaluate to what extent child–juvenile psychopathy could be an extremely relevant correlate in serial murders.

In short, what we can say so far is that there is no specific profile for serial killers, as they are a heterogeneous group of violent delinquents presenting very varied psychosocial and criminological factors [87], some of which can be considered risk factors in this type of multicide [28].

Thus, the aetiology of the psychopathic serial killer is diverse, and its origins are usually evident from numerous serial behaviour patterns in childhood and adolescence [9]. Although the role of psychopathy in serial murders can help police investigations by create profiles [76], further research is needed to help clarify the frequency and degree with which psychopathy is present in serial killers. This would help in the creation of more refined criminal profiles aimed at identifying this type of multicide [83].

## 4. Conclusions

First of all, this review has tried to distinguish between two particularly controversial phenomena that reflect the current landscape of psychosocial and criminological reality: serial killer children and young people, and non-serial killer children and young people. We have tried to focus on the areas that deserve special attention, and we have focused on the extent and seriousness of the problem. The homicides perpetrated by children and young people point to a social panorama that is alarming due to the young age of these murderers. This problem is important enough to warrant study because it “raises exploratory questions about the social, psychological and biological factors that might explain such a tragic phenomenon” [88].

Secondly, the conceptualization of serial murders is still controversial in the sense that it depends on the number of victims that each author stipulates as necessary to be considered as such. In any case, the characterization of this phenomenon is clear, as it is reflected in the criminal profiles of this type of serial killer and what distinguishes them from other forms of multicide (such as mass murder or spree killing). An additional controversy is the enormous media attention surrounding serial murder [89], an effect that is even greater when the perpetrators of the serial murders are minors.

Thirdly, as for the phenomenon of serial murders perpetrated by minors, the extent of the problem is controversial because it often depends on how the number of real cases is counted, while, additionally, the official statistics are difficult to find [90]. 

It can be established that, despite the low prevalence of juvenile serial killers, childhood is a period in which antisocial behaviour begins to flourish. Similarly, the low incidence of serial killers who are minors may be due to the early imprisonment of minors and adolescents who commit a single murder, but who were quite likely to have turned into serial killers [4]. 

On the other hand, although there would seem to be a greater number of adult serial killers, some authors believe it not infrequent for the first murder of this kind to occur in adolescence [36].

In any case, the profiles of children and youths who commit murder are clearly established [91] and, however inconceivable their crimes may seem to us [92], the criminological reality of these minors as murderers is evident, with serious social and legal repercussions [93].

Fourthly, it is also important to consider the psychopathy variable as an influential factor in the diverse modalities of serial criminal conduct committed by children and youths which could be the first ‘steps’ in their criminal career as serial killers later on [9]. In addition to this reason, the importance of the psychopathy variable has also been stressed as a determinant in the process of psychological–criminal profiling of the diverse types of violent serial delinquents, in particular, those who commit sexually aggressive crimes and murder [94].

This current review article is not exempt from limitations. The low prevalence of juvenile serial killers, as well as the presence of juvenile murderers that are not serial killers, makes the generalization of the study more difficult, so the analysis should extend its scope to other serial criminal activities committed by juveniles. Likewise, with respect to the review carried out, it is necessary to comment that a systematic review would have been more valuable.

Despite these limitations, the present review underlines the importance of psychosocial factors for the better understanding of the process by which under-eighteens end up committing such serious crimes as serial murders. We have seen that, on numerous occasions, such crimes are accompanied by other equally serious criminal behaviour patterns (arson, sexual assault, animal abuse, etc.). The research works consulted provide evidence of the special relevance of psychopathy in the generation of serious juvenile delinquency [75].

In this sense, the need to design intervention plans focused on the above-described therapeutic needs is evident. This is because different serial criminal manifestations re-quire specific focuses [94], highlighting the need to deal with these problems with extreme caution in future research.

## Data Availability

Not applicable.
